# Contact Cooling-Induced ELOVL4 Enhances Skin Wound Healing by Promoting the Inflammation-to-Proliferation Phase Transition

**DOI:** 10.7150/ijbs.107871

**Published:** 2025-02-18

**Authors:** Siyi Zhou, Zeming Li, Ke Li, Yuanli Ye, Huan Liang, Nian'Ou Wang, Weiwei Liu, Jingwei Jiang, Martin Y M Chiang, Aijun Chen, Xiao Xiang, Mingxing Lei

**Affiliations:** 1 111 Project Laboratory of Biomechanics and Tissue Repair & Key Laboratory of Biorheological Science and Technology of Ministry of Education, College of Bioengineering, Chongqing University, Chongqing 400044, China.; 2 Shenzhen Accompany Technology Cooperation, Ltd., Shenzhen 518000, China.; 3 Materials Measurement Laboratory, National Institute of Standards and Technology, Gaithersburg, Maryland 20899, United States.; 4 Department of Dermatology, The First Affiliated Hospital of Chongqing Medical University, Chongqing 400042, China.

**Keywords:** wound healing, contact cooling, TNFα, ELOVL4, DHA, EPA

## Abstract

Empirical evidence indicates that the rate of wound healing varies through different seasons, where it is higher in spring and fall but lower in summer and winter, suggesting adequate temperatures may promote wound healing via an unknown mechanism. Here we show that adequate temperature facilitates wound healing by inducing the expression of Elongation of Very Long Chain Fatty Acid Elongase 4 (ELOVL4) that curtails the inflammation phase. Using skin injury and skin organoids models, bulk- and single-cell RNA-sequencing and spatial transcriptomics analysis, and *in vivo* functional perturbations, we first demonstrate that adjusting skin surface temperature to 20°C by contact cooling markedly increases the rate of wound healing via upregulating ELOVL4 in the injured epidermis. We then reveal docosahexaenoic acid (DHA) and eicosapentaenoic acid (EPA) as the key products of ELOVL4 that independently control wound healing by dampening the expression of pro-inflammatory cytokines such as tumor necrosis factor α (TNFα). This chain of physiological events enhances wound healing via its timely exit of the inflammatory phase and entry into the proliferation phase of tissue repair. Our findings highlight the skin's adaptability to different temperatures and link the evolutionarily conserved mechanism of long-chain fatty acid synthesis to wound repair while demonstrating the potential application of contact cooling therapy in wound healing.

## Background

Skin injuries, which are common in daily life, represent pivotal events where the healing assumes a critical role in preserving tissue integrity and function 1, 2. Wound healing constitutes a multifaceted biological phenomenon encompassing various stages, including hemostasis, inflammation, proliferation, and remodeling, which entails a myriad of physiological and molecular processes 3-5. Our previous research has shed light on the role of stem cells in tissue regeneration and the intricate balance between intrinsic cellular properties and extrinsic environmental factors that influence them 6, a finding that may have broader implications for wound healing and tissue repair. Effective wound healing requires the carefully orchestrated interplay of such processes including inflammatory responses, angiogenesis, cell migration, and proliferation 3, 7. Inflammation is a critical stage of wound healing, which coordinates the recruitment and function of immune cells to fend off infection and help establish a conductive milieu for fibroblast and keratinocyte proliferation. However, prolonged inflammatory responses can impede wound healing 8, accentuating the importance of strict and precise control during this process.

Either high temperatures in the summer (37-40°C) or low temperatures in the winter (-10-0°C), are not optimal for wound healing 9. Recent investigations have underscored the significant role of low-temperature (< 37°C) therapy in wound healing due to its remarkable ability to promote the healing of superficial wounds and ease of clinical application (maintaining an ambient temperature within the range of 18-25°C) 10. For instance, the incidence of ulcers, a form of wound-like skin lesion, exhibits a notable spike during warmer seasons (April to October) and clear seasonality 9, indicating a close link between ambient temperature and wound healing. This observation suggests that adequate temperatures may be beneficial for the healing process. For example, daytime radiative cooling (DRC) dressings, which can reduce ambient temperatures by 5-10°C when exposed to sunlight, have been shown to enhance wound healing in mice with full-thickness skin incisions 11. However, how changes in skin surface temperature and the inflammation-proliferation phase transition are coupled to promote wound healing is still largely unclear and requires further investigation.

Some studies have given clues to the above ecophysiological phenomena. The response of skin to different temperature environments is also intricately tied to evolutionary adaptation. During evolution, organisms gradually adapted to diverse environmental temperatures to optimize energy allocation. At lower temperatures, organisms require increased energy to sustain basic physiological functions 12. Exposure to low temperatures typically results in increased energy expenditure, particularly through enhanced fatty acid oxidation to generate additional heat 13. A low-temperature environment can also induce fatty acid synthesis, particularly within the adipose tissue, thereby continuously replenishing energy storage 14. Unsaturated fatty acids, such as eicosapentaenoic acid (EPA) and docosahexaenoic acid (DHA), play crucial roles in modulating inflammatory responses; they can be enzymatically converted into biologically active lipid signaling molecules with anti-inflammatory properties via the COX-2-dependent pathway 15, thereby attenuating inflammation. Moreover, long-chain n-3 polyunsaturated fatty acids can reduce the arachidonic acid content in inflammatory cell membranes, thus lowering the production of pro-inflammatory factors such as prostaglandin E2 (PGE2) 15, 16. Yet, how these biological processes respond to ambient temperature changes and cooperate in harmony to enhance wound healing remains unclear.

In the present study, we show how adequate temperature treatment can markedly promote skin wound healing. We find that contact cooling of the skin to 20°C most effectively promotes wound healing using skin injury models in mice. Transcriptomic analyses revealed that 20°C temperature treatment activates the long-chain fatty acid elongation pathway, particularly involving ELOVL4. This leads to the synthesis and accumulation of eicosapentaenoic acid (EPA) and docosahexaenoic acid (DHA) in the injured epidermis, which significantly downregulates the levels of key pro-inflammatory factors such as tumor necrosis factor α (TNFα). Functional perturbations of ELOVL4-mediated long-chain fatty acid synthesis using small molecule inhibitors delayed wound healing, which was rescued by exogenous EPA or DHA. These findings offer novel insights into the commonly observed seasonality in wound healing by linking, long-chain fatty acid synthesis, an evolutionarily conserved physiological response to different temperatures, to wound healing. Our study also paves the way for the application of such adequate temperature treatment for wound treatment in the clinic.

## Materials and methods

### Mice, wound model, and contact cooling of the skin

The animal experiment protocol has been approved by the Animal Experiment Ethics Committee of Chongqing University. C57BL/6J and nude mice of 8-week-old were purchased from Laibite Biotechnology Company (Chongqing, China). The mice were housed under the following conditions: a stable temperature of 25±1°C, a 12-hour light/12-hour dark cycle, with free access to food and water. To generate the wound model, mice were first anesthetized, then the dorsal hair was removed to expose the bare skin, and after disinfecting the intended wound area, circular cuts with a diameter of 0.6 mm from both flanks of the back. After 1 day of wounding, the mouse wounds and their surrounding skin in a radius of 1.5 cm were subjected to controlled cooling and maintained at 20°C, 10°C, and 6°C using either a hot and cold photon beauty instrument (#B205, BOSEA, China) or a contact cooling instrument (#BSC301/05, PHILIPS, China). The treatment was performed every other day for 2 minutes each time. During wound healing, the following small molecule drugs and recombinant protein were administered via subcutaneous injection to the wound: DHA (MCE, HY-B2167, 200 μmol, 50μL), EPA (MCE, HY-B0660, 200 μmol, 50μL), 2-Chloroacetamide-Inhibitor of ELOVLL4 (MCE, HY-W010629, 50 μmol, 50μL), TNFα (MCE, HY-P7090, 400 μmol, 50μL).

### Bulk RNA-sequencing

We collected skin samples from control groups at PWD4, as well as from 20°C contact cooling treatment groups at PWD4. These PWD4 skin samples were subjected to high-throughput transcriptome sequencing. Library construction and sequencing were performed at Shanghai Majorbio Bio-pharm Biotechnology Co., Ltd. (Shanghai, China) according to the manufacturer's instructions (Illumina, San Diego, CA).

### Transcriptome data analysis

Bulk RNA sequencing data of wound healing at different time points, downloaded from the literature 17, and 20°C treatment bulk RNA-sequencing data were analyzed on the Majorbio Cloud platform (www.majorbio.com). Single-cell RNA-sequencing data for skin without wound and on the third day post-wound were downloaded from the gene ontology (GEO) database (GSE142471). We reanalyzed the data using the statistical software R (version 4.2.0). The R package Seurat version 4.1.3 was used for quality control, cell selection, normalization, identification of highly variable genes, and linear dimensionality reduction. Subsequently, cell marker genes from the literature were referenced for annotation and data preprocessing. Kyoto Encyclopedia of Genes and Genomes (KEGG) enrichment analysis was conducted on the online data analysis and visualization platform at https://www.bioinformatics.com.cn. Spatial transcriptome data for skin without wound and on the third day post-wound were retrieved from the GEO database (GSE166948). The 'hdf5r' function was used to read and write data in HDF5 format. The SCTransform function was used for data normalization to correct technical biases and heterogeneity present in the dataset. The SpatialFeaturePlot function was used to visualize the spatial distribution of target genes, providing a deeper understanding of their expression patterns within the tissue.

### Immunofluorescence

The tissue sections were dewaxed, rehydrated, and then incubated in 10 mM sodium citrate buffer (pH 6.0) at 100°C for antigen retrieval. The slides were blocked with 1% BSA buffer at 37°C for 1 hour, incubated overnight with the primary antibody at 4°C, and then incubated with the secondary antibody at 37°C for 2 hours before visualization under an inverted confocal microscope (Leica, Germany) at the Analysis and Testing Center of Chongqing University. The following antibodies were used in the study: K14 (Boster, BM4522), VIM (BEYOTIME, AF0318), ELOVL4 (Abclonal, A3639), PCNA (Elabscience, E-AB-22001), CD31 (Servicebio, GB12063-100), TNFα (BEYOTIME, AF8208), P63 (Zenbio, 381215), IL1β (BEYOTIME, AF7209). All primary antibodies were diluted at 1:150, and the secondary antibody at 1:500.

### Skin organoid culture

Mouse organoid culture was established as described in our previous studies 18-20. Briefly, the dorsal skin was surgically removed from newborn mice within 24 hours after birth and then floated overnight at 4°C in a 0.25% trypsin solution to separate the dermis and epidermis. The epidermis was further minced with scissors, filtered, and centrifuged to obtain the individual cells. The dermal cells were digested with 0.35% Collagenase I for 20 minutes, followed by filtration and centrifugation. The individual epidermal and dermal cells were mixed at a ratio of 1:9 and seeded into the upper chamber of a Transwell culture system, with 700 μL of DMEM/F12 medium containing 10% FBS added to the lower chamber. The cells were cultured in a 37°C incubator with 5% CO_2_, and the medium was changed every two days.

### Transplantation

Primary dermal/epidermal cell cultures of different treatments were grafted to the dorsal skin of nude mice using our previously developed method 18-20. In brief, a circular wound with a diameter of 1 cm was created from both flanks of the back of nude mice. The organoid culture on the Transwell membrane was flipped onto the wound with the cell side facing down. The membrane was then sutured onto the dorsal skin for fixation and then wrapped with bandages. The bandages were removed four days after transplantation, and the status of wound healing was recorded.

### Enzyme-Linked Immunosorbent Assay (ELISA)

The skin tissue from the wound edge was extracted using a mixture of butanol, methanol, and water in a ratio of 5:25:70 to be used as an analyte. ELISA (Coibo, CB11820-Mu) (Coibo, CB15575-Mu) was performed per the manufacturer's recommended protocol. Briefly, 10 μL of the analyte was mixed with 40 μL of dilution buffer and 50 μL of enzyme-labeled reagent in a 96-well plate. The plate was incubated at 37°C for 60 minutes, washed, and then incubated with 100 μL of substrate solution in the dark at 37°C for 15 minutes. The reaction was terminated with 50 μL of stop solution before being read on a standard microplate reader at 450 nm.

### Quantitative reverse transcription PCR (RT-qPCR)

Total RNA from mouse skin samples and mouse cell organoid cultures was collected using Trizol reagent (#NR0002, Leagene, China) according to the manufacturer's instructions. Then, the RNA was reverse-transcribed to cDNA using the ReverTra Ace RT-qPCR kit (#RR047Q, Takara, Japan) following the manufacturer's protocol. qPCR was performed using the SYBR Green PCR Master Mix (#RR820A, Takara, Japan) with Gapdh as an internal control. The relative gene expression was determined using the ΔΔCT method. Primer sequences used are as follows: Tnfa (Forward: 5' CCCTCACACTCAGATCATCTTCT 3'; Reverse: 5' GCTACGACGTGGGCTACAG 3'), Il1b (Forward: 5' GCAACTGTTCCTGAACTCAACT 3'; Reverse: 5' ATCTTTTGGGGTCCGTCAACT 3'), Il4 (Forward: 5' GGTCTCAACCCCCAGCTAGT 3'; Reverse: 5' GCCGATGATCTCTCTCAAGTGAT 3'), Il6 (Forward: 5' TAGTCCTTCCTACCCCAATTTCC 3'; Reverse: 5' TTGGTCCTTAGCCACTCCTTC 3').

### Statistical analysis

Data are displayed as the average value with the standard error of the mean (SEM), derived from a minimum of three independent experiments. Two-tailed, unpaired Student's t-test was used to compare the sample means. The statistical analysis was conducted using a one-way analysis of variance (ANOVA) to assess the differences among group means. When ANOVA indicates a significant effect, suggesting that at least one group mean differs from the others, Tukey's honestly significant difference (HSD) test is applied as a post hoc analysis. A p-value below 0.05 was considered statistically significant. All statistical analyses were performed using GraphPad Prism 8 software.

## Results

### Contact cooling of the skin to 20°C promotes skin wound healing

**Figure 1:**
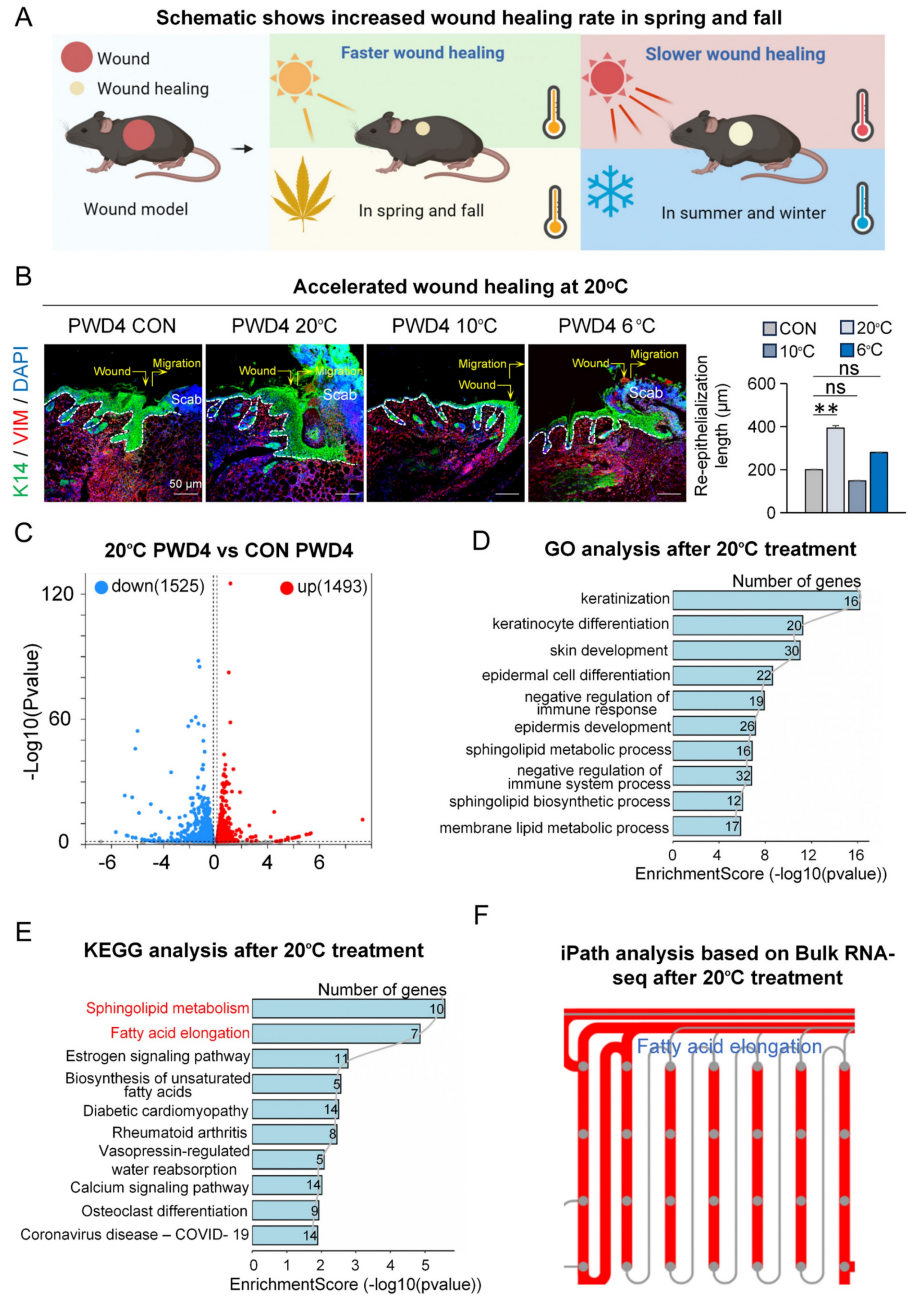
**20°C treatment promotes skin wound healing.** A. Schematic of wound healing rate in different seasons. B. K14 and VIM immunostaining shows 20°C treatment promotes wound healing. Scale bars, 50μm. Statistical of the length of the regenerated epidermis. N ≥ 5, **p < 0.01, ns: no significance. C. Volcano Plot shows different expressed genes in the 20°C-treatment group and control group. D. GO shows skin development pathway enriched in the 20°C-treatment group. E. KEGG shows fatty acid elongation pathway enriched in the 20°C-treatment group. F. iPATH shows fatty acid elongation pathway increased after 20°C treatment.

To explore if and how adequate temperature treatment influences skin wound healing (Figure 1A), we first assessed wound healing after contact cooling to various temperatures (6°C, 10°C, and 20°C) by immunofluorescence staining at post-wounded day 4 (PWD4). We observed significantly increased re-epithelialization and dermal cell migration, indicating accelerated wound healing, particularly after contact cooling to 20°C, compared to the control at 37°C 21 (Figure 1B), suggesting contact cooling as a potentially effective strategy to aid wound closure.

To investigate the potential mechanism by which 20°C treatment promotes wound healing, we performed bulk RNA sequencing and compared the transcriptomic changes between the 20°C-treatment group and the controls four days after wounding (Figure S1A). The volcano plot results showed 1,493 upregulated genes and 1,525 downregulated genes in the 20°C-treatment group compared to the control (Figure 1C). Gene Ontology (GO) analysis on the upregulated genes in the 20°C-treatment group revealed significant enrichment in pathways relating to keratinization, skin development, and negative regulation of immune response (Figure 1D). This suggested that after 20°C treatment, the re-epithelialization was enhanced whereas the immune response was suppressed in the wound bed, which is corroborated with our STRING analysis results that showed significant enrichment of genes associated with re-epithelialization (Figure S1B). Interestingly, KEGG analysis revealed that the differentially upregulated genes were enriched in pathways such as sphingolipid metabolism and fatty acid elongation (Figure 1E), which was also in accordance with the results from iPATH4.0 analysis (Figure 1F). This implies that the metabolic process of long-chain fatty acids (LCFAs) may be regulated by contact cooling treatment during wound healing.

### Increased LCFAs after contact cooling treatment

**Figure 2:**
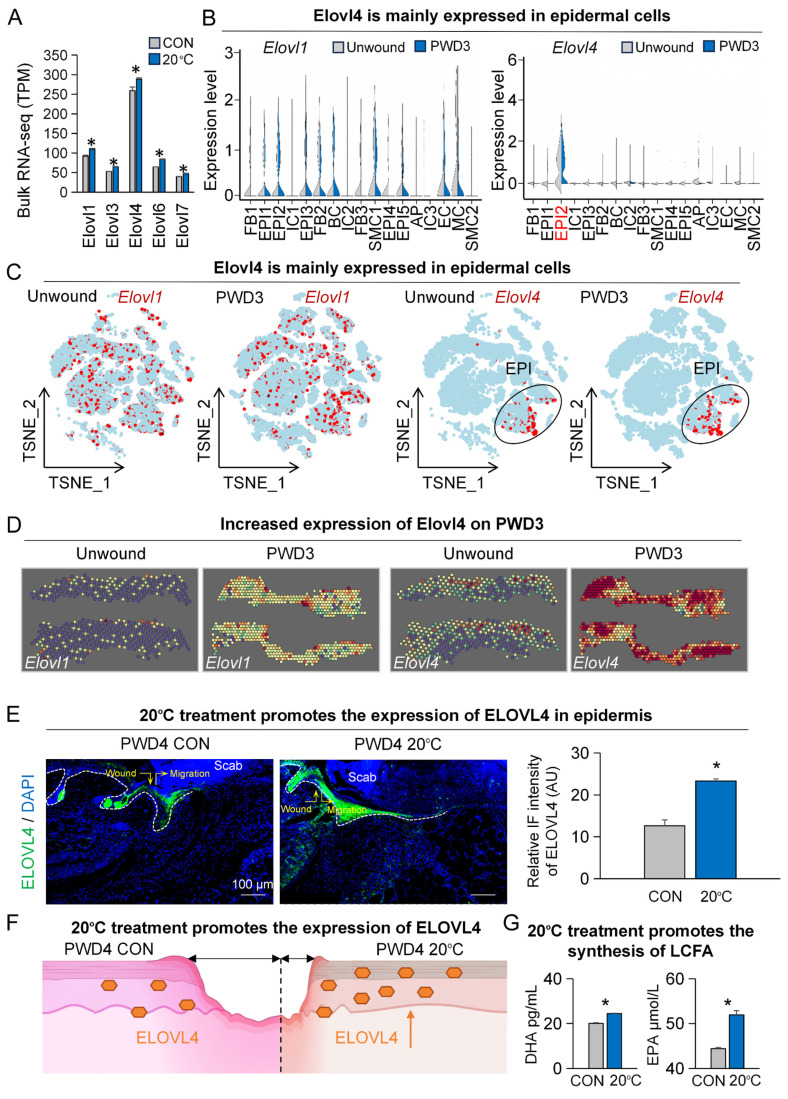
** Low temperatures promote the expression of ELOVL4 during wound healing.** A. Bulk RNA-seq shows expression of Elovl-family genes increased in the 20°C-treatment group. N ≥ 5, *p < 0.05. B. VlnPlots show expression of Elovl1 and Elovl4 in unwound and PWD3 skin. C. FeaturePlots show expression of Elovl1 and Elovl4 in unwound and PWD3 skin. D. ST-seq shows expression of Elovl1 and Elovl4 in unwound and PWD3 skin. E. ELOVL4 immunostaining shows increased expression in the 20°C-treatment group. Statistics of average FI of ELOVL4. Scale bars, 100μm. N ≥ 5, *p < 0.05. F. Schematic shows increased expression of ELOVL4 in the 20°C-treatment group. G. ELISA shows DHA and EPA were increased in the 20°C-treatment group. N ≥ 5, *p < 0.05.

In response to low ambient temperature, our body tends to increase LCFA metabolism as an energy source to maintain optimal core temperature 22. To assess the changes in the synthesis levels of LCFAs after contact cooling treatment, we compared the expression of key genes involved in LCFA synthesis including Elovl1, Elovl3, Elovl4, Elovl6, and Elovl7 between the contact cooling treatment and control groups on PWD4 by bulk RNA-sequencing. Surprisingly, all the above-mentioned genes were significantly upregulated after contact cooling treatment (Figure 2A). As Elovl1 and Elovl4 were most highly expressed amongst all tested genes, they were selected for further analysis.

We first analyzed single-cell RNA-sequencing data to examine the expression patterns of Elovl1 and Elovl4 with and without skin wounding (Figure S2A-B). Elovl1 was expressed across various cell populations, with no obvious difference in their expression patterns between the wounded and unwounded groups (Figure 2B-C).

Interestingly, Elovl4 was primarily expressed in the suprabasal epidermal cells (EPI2, Krt1+, and Krt10+, Figure S2B-C), and its expression level was noticeably increased after skin wounding (Figure 2B-C). To further ascertain the expression footprint of Elovl1 and Elovl4 after skin wounding, we analyzed spatial transcriptome data (Figure S2C). Our analyses revealed that the expression of both Elovl1 and Elovl4 were upregulated at PWD3, particularly Elovl4 is greatly increased in and near the wound bed, further suggesting its potential function in wound healing (Figure 2D).

As we observed a more pronounced increase in Elovl4 levels (Figure 2D), we selected it for downstream studies. As such, we began by assessing the expression of ELOVL4 in both the contact cooling treatment and control groups on PWD3 by immunofluorescence, which confirmed that ELOVL4 was predominantly expressed in the suprabasal layer of the epidermis and its expression was significantly increased after contact cooling treatment compared to the control group (Figure 2E-F). To further investigate whether the content of LCFAs changed after contact cooling treatment, we measured the relative levels of DHA and EPA using a metabolic assay kit and observed that the DHA and EPA were both significantly higher in the 20°C-treatment group compared to the control (Figure 2G). These findings suggest that 20°C treatment during wound healing can upregulate the expression of ELOVL4, thereby driving the synthesis of long-chain fatty acids such as DHA and EPA.

### Elovl4-EPA/DHA promotes wound healing

**Figure 3:**
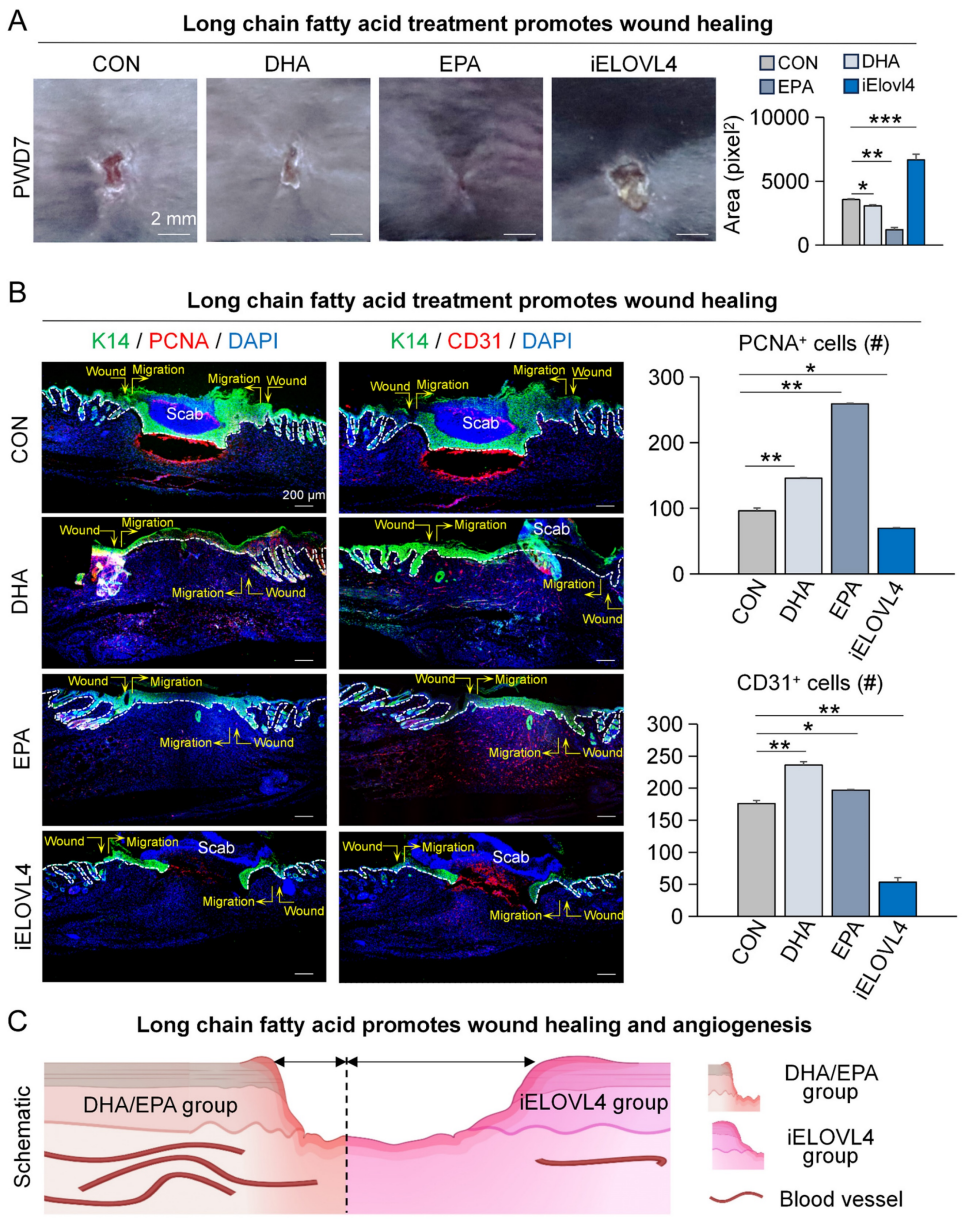
** Elovl4-EPA/DHA promotes wound healing.** A. Phase-contrast microscope and statistics show wound healing after long chain fatty acid treatment; Statistics of average wound size. Scale bars, 2 mm. N ≥ 5, ***p < 0.001, **p < 0.01, *p < 0.05. B. K14/PCNA and K14/CD31 immunostaining shows wound healing after DHA, EPA, or ELOVL4 inhibitor treatment; Statistics of average PCNA^+^ and CD31^+^ cells. Scale bars, 200μm. N ≥ 5, **p < 0.01, *p < 0.05. C. Schematic shows wound healing after DHA, EPA, or ELOVL4 inhibitor treatment.

While EPA and DHA have been implicated in the inflammatory response, their role in wound healing has not been fully elucidated. Although some studies suggest potential benefits, the specific mechanisms by which these fatty acids promote wound healing are not yet clear 23, 24. Thus, we treated the wounded skin with DHA, EPA, and 2-Chloroacetamide (an inhibitor of ELOVL4) on PWD0, 2, 4, and 6, and the samples were collected on PWD7 for analysis (Figure S3A). The results showed that the DHA- and EPA-treated groups exhibited significantly faster wound healing, whereas inhibition of ELOVL4 (iELOVL4) caused a clear delay in wound healing compared to the control group (Figure 3A). K14 and Proliferating Cell Nuclear Antigen (PCNA) labeling of skin wound cross-sections indicated that the wounds with DHA or EPA treatment were completely re-epithelialized, with tighter connections between epidermis and dermis compared to the control, a clear indication of enhanced wound healing. In contrast, the iELOVL4 group showed worse wound healing evident by the presence of the large scab and incomplete re-epithelialization underneath (Figure 3B).

Moreover, angiogenesis can also serve as an indicator of the state of wound healing 25, 26. Thus, we immunolabeled the same skin wound cross-sections for K14 and CD31, markers for epithelial cells and endothelial cells, respectively. The results showed that the number of vascular endothelial cells was significantly higher in groups that received DHA and EPA compared to control, while it was significantly lower in the iELOVL4 group (Figure 3B). Collectively, these results suggest that LCFAs can accelerate wound healing by inducing re-epithelialization and angiogenesis (Figure 3C).

### Elovl4-EPA/DHA inhibit TNFα in wound healing

**Figure 4:**
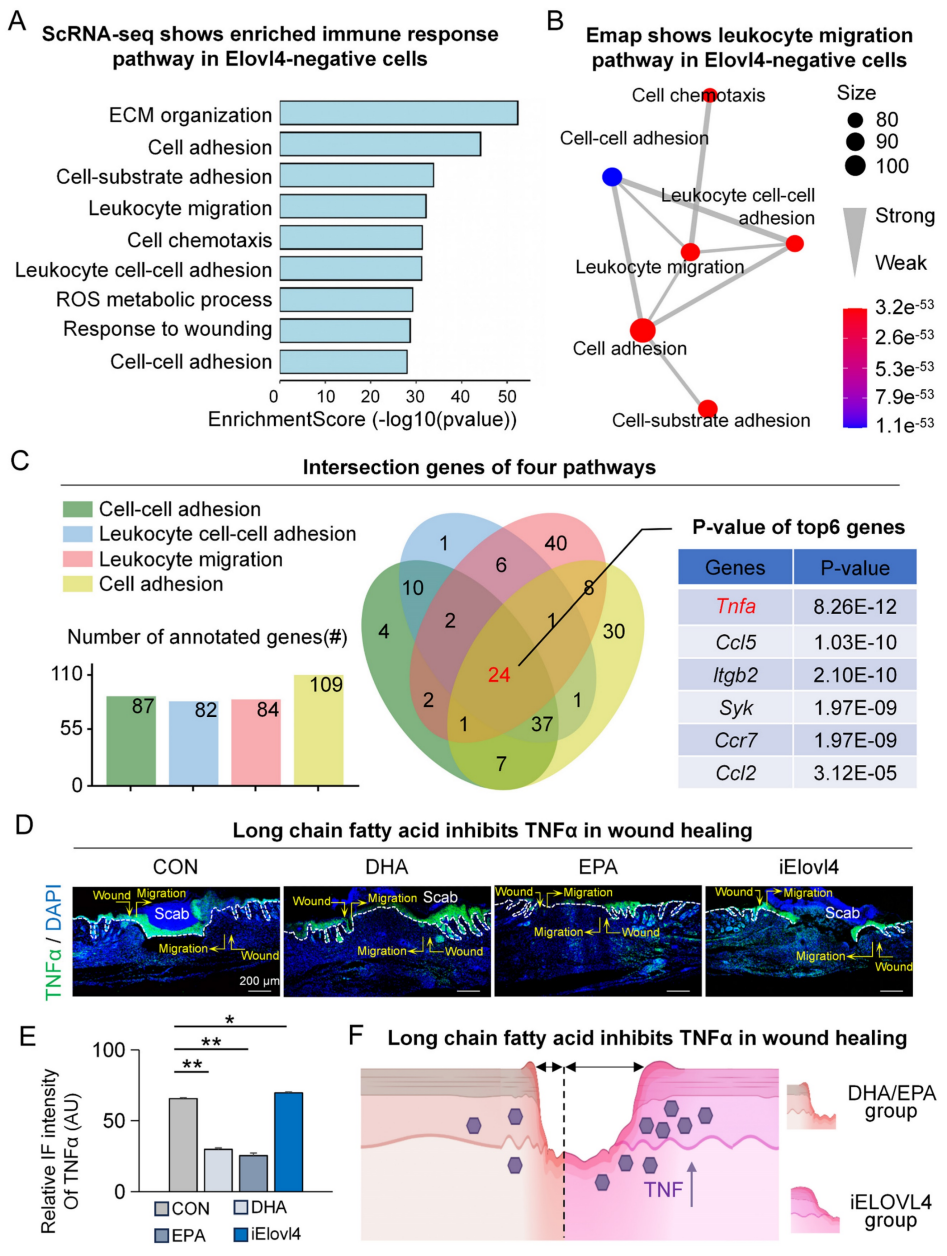
** Elovl4-EPA/DHA inhibit TNFα in wound healing.** A. GO shows that the immune response pathway was enriched in Elovl4 negative cells. B. Emap shows that the leukocyte migration pathway was enriched in the center. C. Venn Plot shows that TNFα was enriched. D. Immunostaining shows the expression of TNFα in the control, DHA, EPA, and iElovl4 groups. Scale bars, 50μm. E. Schematic shows DHA/EPA inhibit TNFα in wound healing.

Next, we explored the mechanism by which ELOVL4 and its LCFAs, EPA, and DHA (ELOVL4-EPA/DHA) promote wound healing. We started by comparing the differentially expressed genes between ELOVL4-positive and ELOVL4-negative cells in the single-cell RNA-sequencing data. The results showed that pathways such as cell adhesion, leukocyte migration, and cell chemotaxis were highly enriched (Figure 4A) with leukocyte migration being in the center of the network of the enriched pathways (Figure 4B), suggesting the potential involvement of ELVOVL4 in the immune response during wound healing. We then utilized Venn interaction analysis to examine the enriched pathway genes which showed that the 24 genes were common in all four pathways (Figure 4C), with Tnfa, encoding tumor necrosis factor α (TNFα) ranked highest (Figure S4A-B). To confirm this, we assessed the levels of TNFα in the wound bed by immunofluorescence. Consistent with our bioinformatical analyses, TNFα expression was reduced in the epidermis in both DHA- and EPA-treated groups compared to the control group, while it was significantly increased in the iELOVL4 group (Figure 4D-E). These results indicate that ELOVL4 may promote wound healing by suppressing TNFα-mediated inflammatory responses (Figure 4F).

### Exposure to adequate contact cooling can inhibit TNFα-mediated inflammatory response

**Figure 5:**
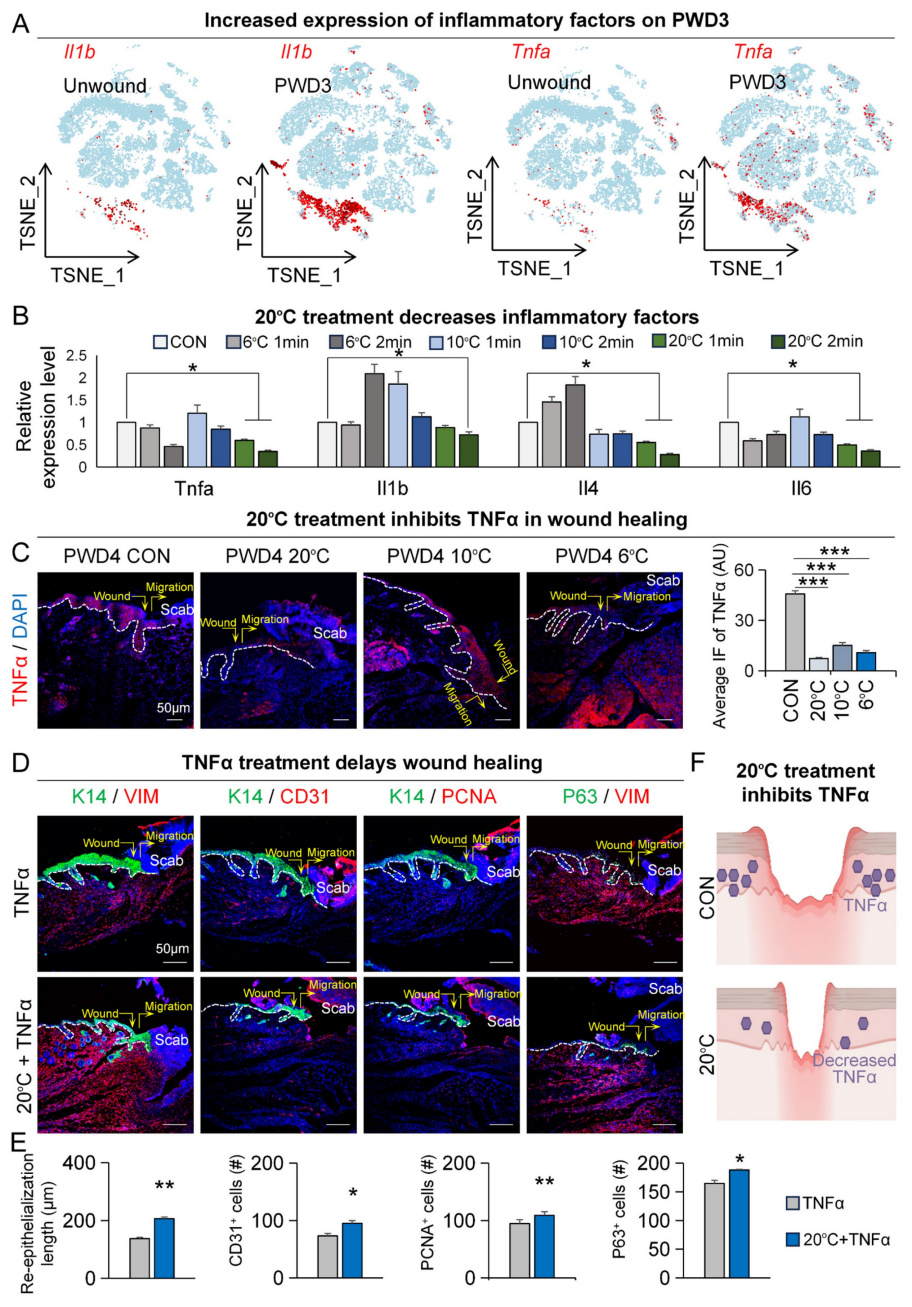
** 20°C treatment can inhibit TNFα-mediated inflammatory response.** A. FeaturePlots show increased expression of Il1b and Tnfa in PWD3. B. qRT-PCR shows the expression of inflammatory factors decreased in the 20°C group. N ≥ 5, **p < 0.01, ***p < 0.001, ns: no significance. C. TNFα immunostaining shows the decreased expression of TNFα in the 20°C-treatment group; Statistics of average FI of TNFα. Scale bars, 100μm. N ≥ 5, **p < 0.01, *p < 0.05. D. K14/VIM, K14/CD31, K14/PCNA, and P63/VIM immunostaining shows wound healing in the TNFα group and the 20°C treatment +TNFα group. E. Statistics of the average rate of re-epithelialization. Scale bars, 100μm. N ≥ 5, **p < 0.01, *p < 0.05. F. Schematic shows decreased expression of TNFα after 20°C treatment.

As our previous GO analysis revealed a notable enrichment of negative regulation of immune responses following contact cooling treatment (Figure 1D), we wondered whether other important inflammatory factors than TNFα were modulated by the 20°C treatment. Therefore, we analyzed single-cell RNA-sequencing data and spatial transcriptome data for these changes. The FeaturePlot of single-cell RNA-sequencing data showed that, compared to the unwound group, the expression of inflammatory factors Il1b and Tnfa increased on PWD3 (Figure 5A).

Spatial transcriptome results also showed increased expression of multiple inflammatory factors on PWD3 at the wound site (Figure S5A). To further explore the association between low temperature and inflammation in wound healing, we profiled the change in expression of various inflammatory factors on PWD1 to PWD3 after 20°C treatment. Notably, qRT-PCR showed that 20°C treatment caused consistent downregulation of inflammatory factors, such as Tnfa, Il1b, and Il6 (Figure 5B). Intriguingly, treatment with lower temperatures, such as 10°C or 6°C, instead increased the expression of some inflammatory factors like Il1b and Il4 (Figure 5B). Nevertheless, immunofluorescence staining of TNFα in the skin wound cross-sections further confirmed its significant reduction after treatment at both 20°C, 10°C and 6°C treatment compared to the control (37°C) 21 (Figure 5C). This suggests that injury-induced inflammatory response is subject to spatiotemporal tuning by ambient temperatures.

We then investigated whether prolonged or elevated TNFα expression can inhibit wound healing. To gain a more comprehensive understanding, we conducted enrichment analysis on both TNFα-positive and TNFα-negative epidermal cells within the PWD3 sample using single-cell RNA-sequencing data. The GO enrichment analysis results indicated that pathways associated with epidermal development were significantly enriched in TNFα-negative cells compared to TNFα-positive cells (Figure S5B). Additionally, KEGG enrichment analysis revealed that inflammatory signaling pathways were significantly enriched in TNFα-positive cells, while lipid metabolism pathways were ranked higher in TNFα-negative cells compared to TNFα-positive cells (Figure S5B). These enrichment analyses strengthened the link between TNFα, wound healing, and lipid metabolism. Next, we assessed the function of TNFα during wound healing. Our immunofluorescence results showed that, compared to treatment with TNFα alone, 20°C treatment accelerated wound healing in the presence of TNFα by showing increased re-epithelialization (K14/VIM), vascular endothelial numbers (K14/CD31), number of proliferating cells (K14/PCNA), and epithelial regeneration (K14/P63) (Figure 5D-E). Together, these results suggest that low adequate temperature promotes skin wound healing by suppressing the expression of TNFα (Figure 5F).

### Elovl4-EPA/DHA promotes skin organoids to regenerate skin

**Figure 6:**
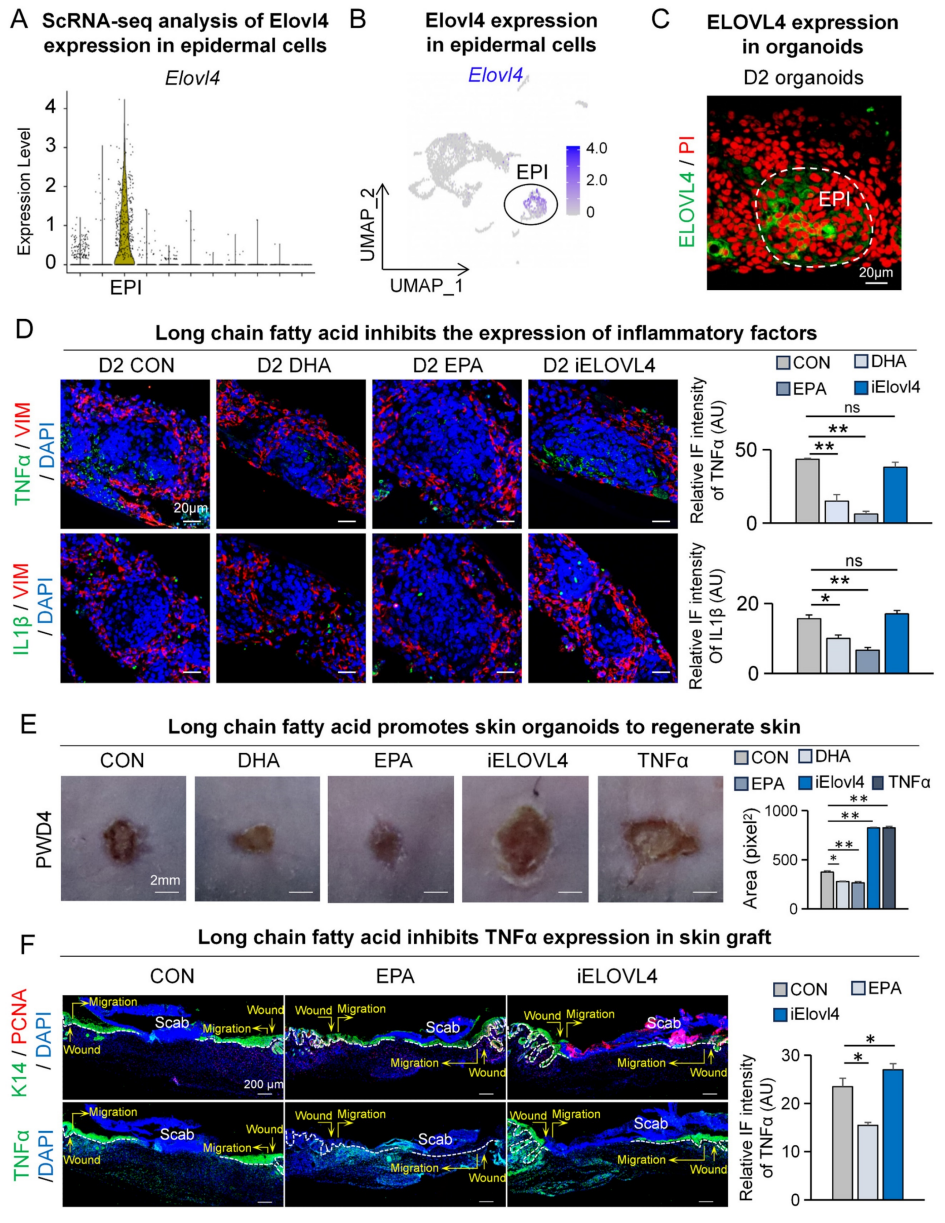
** Elovl4-EPA/DHA promotes skin organoids to regenerate skin.** A. VlnPlot shows expression of Elovl4. B. FeaturePlot shows Elovl4 expressed in epidermal cells. C. Immunostaining shows that ELOVL4 is expressed in epidermal cells. D. TNFα/VIM and IL1β/VIM immunostaining shows the decreased expression of inflammatory factors after LCFAs treatment; Statistics of average FI of TNFα- and IL1β-expressing cells. Scale bars, 20μm. N ≥ 5, **p < 0.01, *p < 0.05, ns: no significance. E. Phase-contrast microscope and statistics show wound healing after long chain fatty acid treatment; Statistics of average wound size. N ≥ 5, **p < 0.01, *p < 0.05. F. K14 / PCNA and TNFα immunostaining shows depressed wound healing in the iELOVL4 transplantation group. Statistics of PCNA^+^ cells and average FI of TNFα. Scale bars, 100μm. N ≥ 5, *p < 0.05.

Autologous transplantation of tissue-engineered skin substitutes generated using stem cells has received increasing attention in treating patients with moderate to severe skin injuries 18-20, 27-29. To further explore the feasibility of using contact cooling treatment-induced LCFAs to aid wound healing in the aforementioned scenario, we utilized our previously established skin organoids, a potential building block of tissue-engineered skin substitute, for investigation 18-20, 27-29. We first assessed the expression of Elovl4 within skin organoids by analyzing single-cell RNA-sequencing data from the organoids (Figure S6A-B). The VlnPlot and FeaturePlot results showed that ELOVL4 is primarily expressed in the epidermal cells (Figure 6A-B). Concurrently, we performed ELOVL4 immunofluorescence staining on the organoids, which revealed that ELOVL4 is mainly expressed in the suprabasal epidermal cells (Figure 6C).

To assess if the inflammatory response could be recapitulated in skin organoids and modulated by LCFAs, we assessed TNFα and IL1β expression by immunofluorescence. Compared to the control group, the expression of TNFα and IL1β in the epidermis was reduced in the DHA and EPA groups, while the expression significantly increased in the iELOVL4 group (Figure 6D).

Next, we performed a skin organoid culture transplantation assay to verify the function of LCFA in wound healing. Skin organoid cultures were pre-treated with DHA, EPA, and iELOVL4 before being transplanted onto the backs of nude mice. Samples were collected on the fourth day after transplantation to observe the condition of wound healing (Figure S6C). The results from the nude mouse back transplantation showed that compared to the control group, DHA- and EPA-treated groups exhibited accelerated wound healing, whereas the iELOVL4 and TNFα groups showed delayed wound healing similar to previous observations (Figure 6E). We again immunolabelled the skin grafts for PCNA and TNFα four days after transplantation to further assess the condition of wound healing. As expected, our results showed accelerated wound healing after EPA treatment, with more complete re-epithelialization, tight epidermis to dermis connection, and reduced TNFα as seen before, whereas inhibition of ELOVL4 had the opposite effects (Figure 6F). Altogether, skin organoids re-capitulated our previous *in vivo* results and further demonstrated that LCFA can promote skin wound healing by reducing TNFα expression.

## Discussion

An interesting and currently unexplored area in the field of tissue regeneration is the relationship between temperature and wound healing. The effectiveness of wound healing varies across different seasons, with outcomes in spring and fall often being superior to those in summer and winter 9. Here we report that cooling skin wounds to 20°C by direct contact with a device with a cold surface enhances skin wound healing by stimulating ELOVL4-mediated CLFA synthesis. Traditionally, this evolutionary adaptation has been implicated in energy consumption and insulation 30, 31. However, here we further link this process to wound healing as the synthesized CLFAs, EPA and DHA, have profound roles in suppressing injury-induced pro-inflammatory cytokines, thus enhancing healing by promoting timely entry into the proliferation phase from the inflammation phase of wound repair.

Excessively high temperatures in summer (40-45°C) may stimulate and prolong the inflammatory responses at the wound site of various tissues, including skin, thereby delaying the healing process 32. Whereas extremely low temperatures in the winter (< -10°C) may impair skin barrier function, also hindering wound healing 32. We decided to expose the wound area to a range of intermediate temperatures and found that acute exposure cooling to 20°C promoted wound healing most effectively. The skin tissue around the injury exhibits an increase in temperature during the early stages of wound healing, which is closely related to the intense inflammatory response 4, 33. Although early onset of inflammation is not only necessary for fighting against infection, excessive or prolonged inflammation can be detrimental, leading to significant delays in wound healing 34. Thus, this process needs to be tightly regulated. Indeed, it has been well documented that the inflammatory response peaks around 24-36 hours post-injury and then subsides to basal levels 17. In addition, previous evidence has suggested that 4°C treatment can suppress inflammatory responses 35. However, here we demonstrate that accelerating the resolution of the inflammation phase can promote the wound-healing process.

In this study, we observed that adequate low-temperature treatment at 20°C around the wound area had a significant inhibitory effect on inflammation but also facilitated wound healing (Figure 1B, 4D). Moreover, during the natural process of wound healing, the expression of inflammatory cytokines TNFα, IL1α, IL1β, and Cxcl2 reached their peaks around 24 hours post-wounding before subsiding to baseline levels (Figure S7). Interestingly, it is noteworthy that we started applying contact cooling treatment to the wound area a day after wounding, which was at the beginning of the resolution phase of inflammation. Therefore, our results not only are in complete agreement with previous findings, but they also suggest that adequate temperature treatment can hasten the decline of the inflammatory response, and consequently allow the wound to enter the proliferation phase more rapidly. Furthermore, as inflammation plays a complex dichotomic role in wound healing, it is important to engage the wound with optimal temperatures at the favorable time of the healing process to obtain beneficial effects.

**Figure 7:**
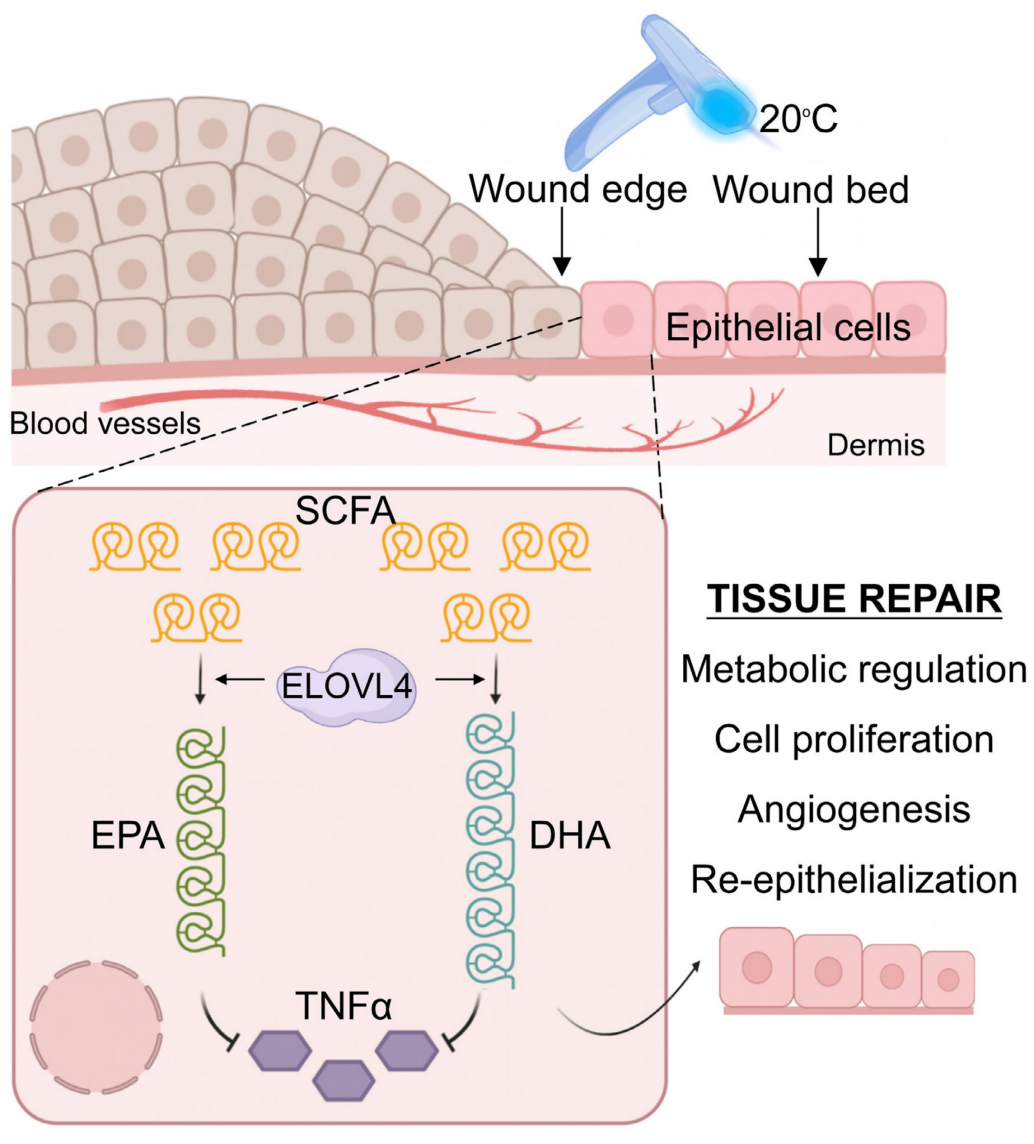
** Graphical abstract.** Contact cooling treatment increases the expression of ELOVL4 in regenerating epithelial cells, thereby promoting the synthesis of long-chain fatty acids including EPA and DHA, which inhibit the expression of TNFα, ultimately enhancing skin wound healing by promoting the inflammation-to-proliferation phase transition.

In addition to suppressing inflammatory responses, a variety of biological processes are regulated by temperature, such as enzyme activity, cell proliferation, and metabolism 36-40. When the body is exposed to a cold environment, it often requires more energy metabolism to maintain homeostasis 41, 42. Through a detailed analysis of the skin transcriptome following adequate temperature treatment and subsequent validations, we demonstrated the significant role of the long-chain fatty acid synthesis pathway following 20°C treatment during wound healing. 20°C treatment induced ELOVL4, which is a key enzyme involved in the synthesis of DHA and EPA, two important omega-3 long-chain polyunsaturated fatty acids. Interestingly, DHA and EPA have been shown to possess anti-inflammatory properties, promote cell proliferation and migration, and regulate cellular signaling in various cell types 23, 43. Even at low temperatures, the expression of ELOVL4 can still be increased, yet it fails to promote wound healing (Figure S2D). At excessively low temperatures like 6°C and 10°C, the fatty acid tails of phospholipids move less and become more rigid 44. This alteration leads to a reduction in the overall fluidity of the membrane and also decreases its permeability. Consequently, it potentially restricts the entry of crucial molecules such as oxygen and glucose into cells. Moreover, according to the studies 45, 46, under these low-temperature conditions, the body's metabolic rate usually drops. This decline may have an impact on the utilization efficiency of fatty acids after synthesis. As a result, despite the increase in synthesis, the actual supply of energy and substances required for the healing process and inflammatory responses does not increase simultaneously. This could be the underlying cause for the slower healing speed of the 6°C and 10°C treatment groups when compared to the 20°C group. Therefore, we hypothesize that the increased synthesis of DHA and EPA mediated by ELOVL4 may be one of the major molecular mechanisms by which contact cooling treatment promotes wound healing (Figure 7). Furthermore, our findings are in line with existing literature on the role of long-chain fatty acids in skin biology. For instance, DHA and EPA have been reported to modulate skin immune responses, reduce the release of inflammatory mediators, and promote the restoration of skin barrier function 47. Specifically, previous studies have shown that EPA and DHA can inhibit the expression of inflammatory cytokines, including TNFα 48-52 which is also recapitulated in our current study where exogenous addition of EPA and DHA significantly reduced the expression of TNFα and improved wound healing.

TNFα, as a pleiotropic inflammatory cytokine, has been extensively studied for its role in regulating immune responses and cell proliferation and has been implicated in various pathological states, including inflammation and autoimmune diseases 53, 54. Through single-cell RNA-sequencing analysis, we found that the expression of TNFα in epithelial cells post-wound increased significantly and was significantly associated with pathways related to immune response and epidermal development. Moreover, our experimental results further confirmed the negative effects of TNFα on skin wound healing, particularly in terms of wound healing speed and neovascularization. Interestingly, ELOVL4 and TNFα are both mainly found in the epidermis and such co-localization in skin also suggests a chain of biological responses where contact cooling treatment induces the expression of ELOVL4, a metabolic sensor, resulting in elevated production of EPA and DHA in the epidermis, which leads to an overall reduction in TNFα expression, accelerated resolution of inflammation, and ultimately improved wound healing speed. Although the exact mechanism of how EPA and DHA inhibit TNFα requires further investigation, their co-expression in the epidermis suggests a potential inhibitory function of EPA and DHA or their metabolites in TNFα-producing immune cells. Nevertheless, our data make adequate temperature as well as DHA and EPA potential therapeutic options for various skin conditions, including inflammatory skin diseases and wound-healing disorders such as psoriasis and keloid, respectively.

Despite the strong evidence provided by our study on the wound healing-promoting effects of 20°C treatment, there are some limitations that warrant further exploration. For instance, we mainly focused on the direct impact of low-temperature treatment on the inflammatory response, with less exploration of other potential factors such as angiogenesis and extracellular matrix remodeling, which happen at the later stages of wound healing 55. We have yet to investigate whether low temperature can modulate inflammation via organelles as numerous studies have demonstrated that the level of mitochondrial oxidative stress is closely associated with inflammation 56. In this study, we focused on the regulation of TNF-α by low temperatures. Other inflammatory factors such as COX-2 also play crucial roles in wound healing 57, which warrants further investigation. Furthermore, this study was primarily conducted in a mouse model, and future research is needed to further validate these findings in human skin samples.

## Conclusions

Our study provides new insights into the application of adequate temperature treatment by contact cooling in promoting wound healing and reveals the important role of long-chain fatty acids in wound healing and addresses a long-standing question of how fluctuating temperatures influence wound healing at different seasons. Future research can build on these findings to further explore the synergistic effects of cold and fatty acids in skin wound healing and their underlying molecular mechanisms.

## Supplementary Material

Supplementary figures.

## Abbreviations

ARA: Arachidonic acid
BSA: Bovine serum albumin
COX-2: Cyclooxygenase-2
DAPI: 4'6'-diamidino-2-phenylindole
DHA: Docosahexaenoic acid
DRC: Daytime radiative cooling
KEGG: Kyoto encyclopedia of genes and genomes
ELISA: Enzyme-linked immunosorbent assay
ELOVL4: Elongation of very long chain fatty acids protein 4
EPA: Eicosapentaenoic acid
GEO: Gene ontology
iELOVL4: Inhibitor of ELOVL4
IL-1β: Interleukin 1 beta
Krt1: Keratin 1
Krt14: Keratin 14
Krt25: Keratin 25
Krt71: Keratin 71
Krt73: Keratin 73
LCFAs: Long-chain fatty acid
OD: Optical density
PCNA: Proliferating cell nuclear antigen
PGE2: Prostaglandin E2
PWD3: Post wound day 3
P63: Tumor protein p63
qRT-PCR: Real-time quantitative reverse transcription PCR
SCFA: Short-chain fatty acids
TNFα: Tumor necrosis factor A
VIM: Vimentin
