# Supplementary material for: Contact Cooling-Induced ELOVL4 Enhances Skin Wound Healing by Promoting the Inflammation-to-Proliferation Phase Transition
Source: Int J Biol Sci. 2025 Feb 18;21(5):2067–82. doi: 10.7150/ijbs.107871 (PMC11900824; doi:10.7150/ijbs.107871)
Supplement: Supplementary file 1 — Supplementary figures. [file ijbsv21p2067s1.pdf]

Supplementary materials

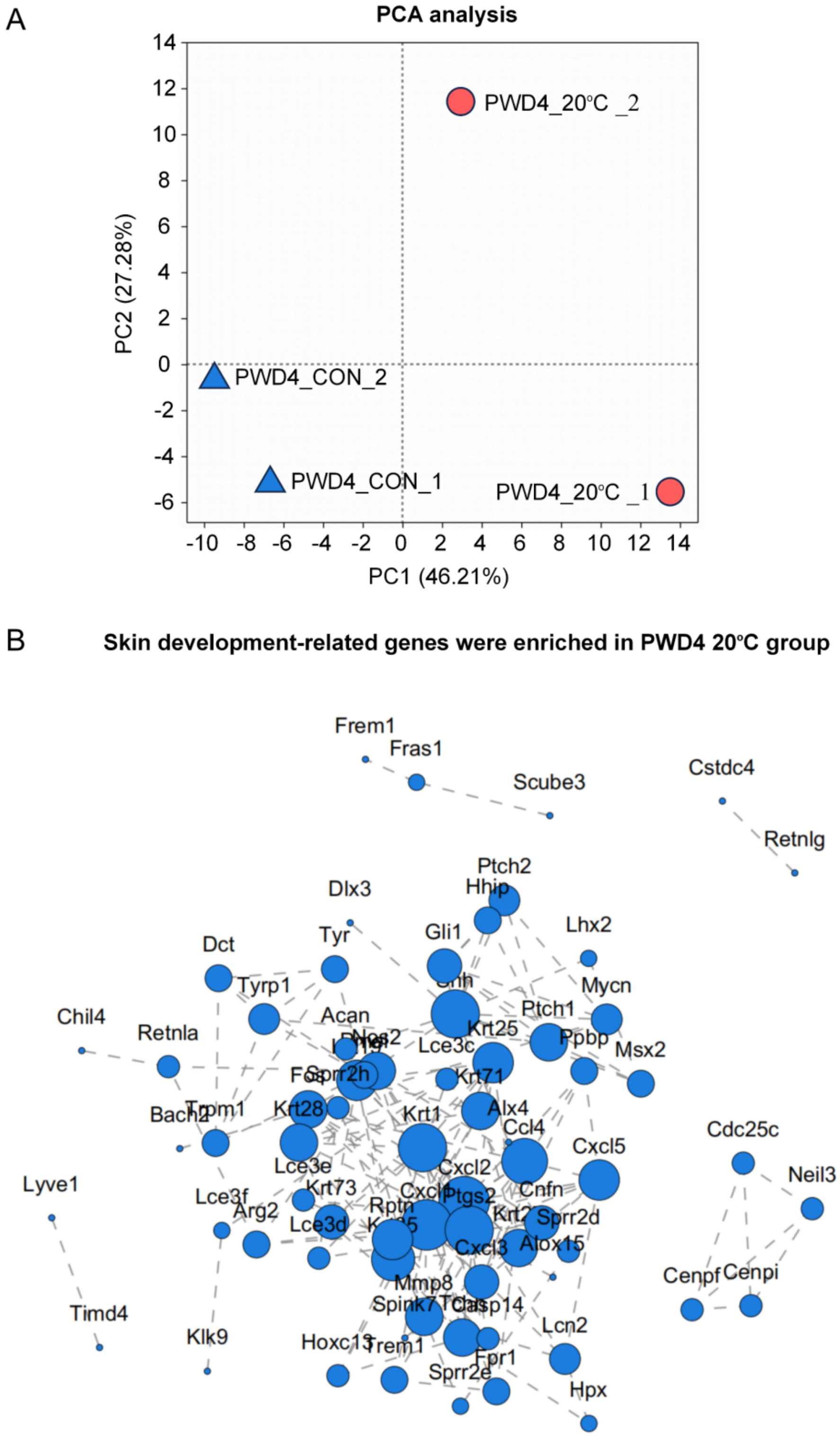

Figure S1. 20℃ treatment promotes wound healing.

A. PCA reveals the differences between the PWD4 CON and PWD4 20℃ treatment samples.

B. String analysis shows skin development-related genes were enriched in the 20℃-treatment group on PWD4.

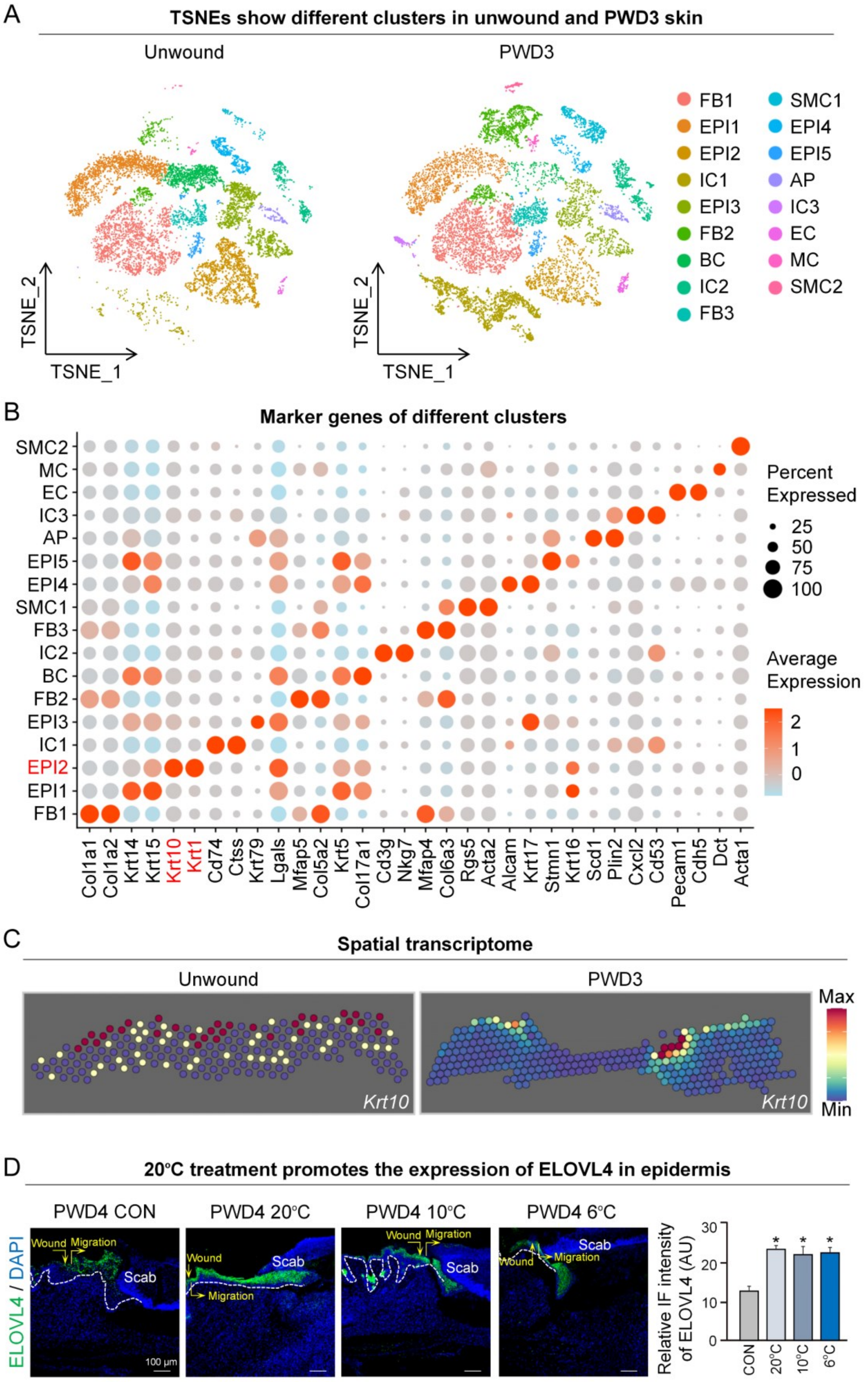

Figure S2. Single cell RNA-sequencing analysis of skin wound.

A. TSNEs show different clusters in the unwound and PWD3 groups.

B. DotPlot shows marker genes of different clusters.

C. ST-seq shows marker genes of the epidermis.

D. Immunostaining shows ELOVL4 expression in the Control, 20°C-treatment, 10°C-treatment, and 6°C-treatment group. Statistics of average FI of ELOVL4. Scale bars, 100μm. $N \geq 5$, $^*p < 0.05$.

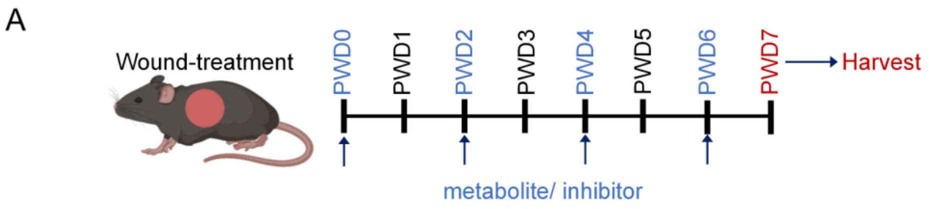

Figure S3. Functional study of Elovl4-EPA/DHA.

A. Schematic shows experimental design.

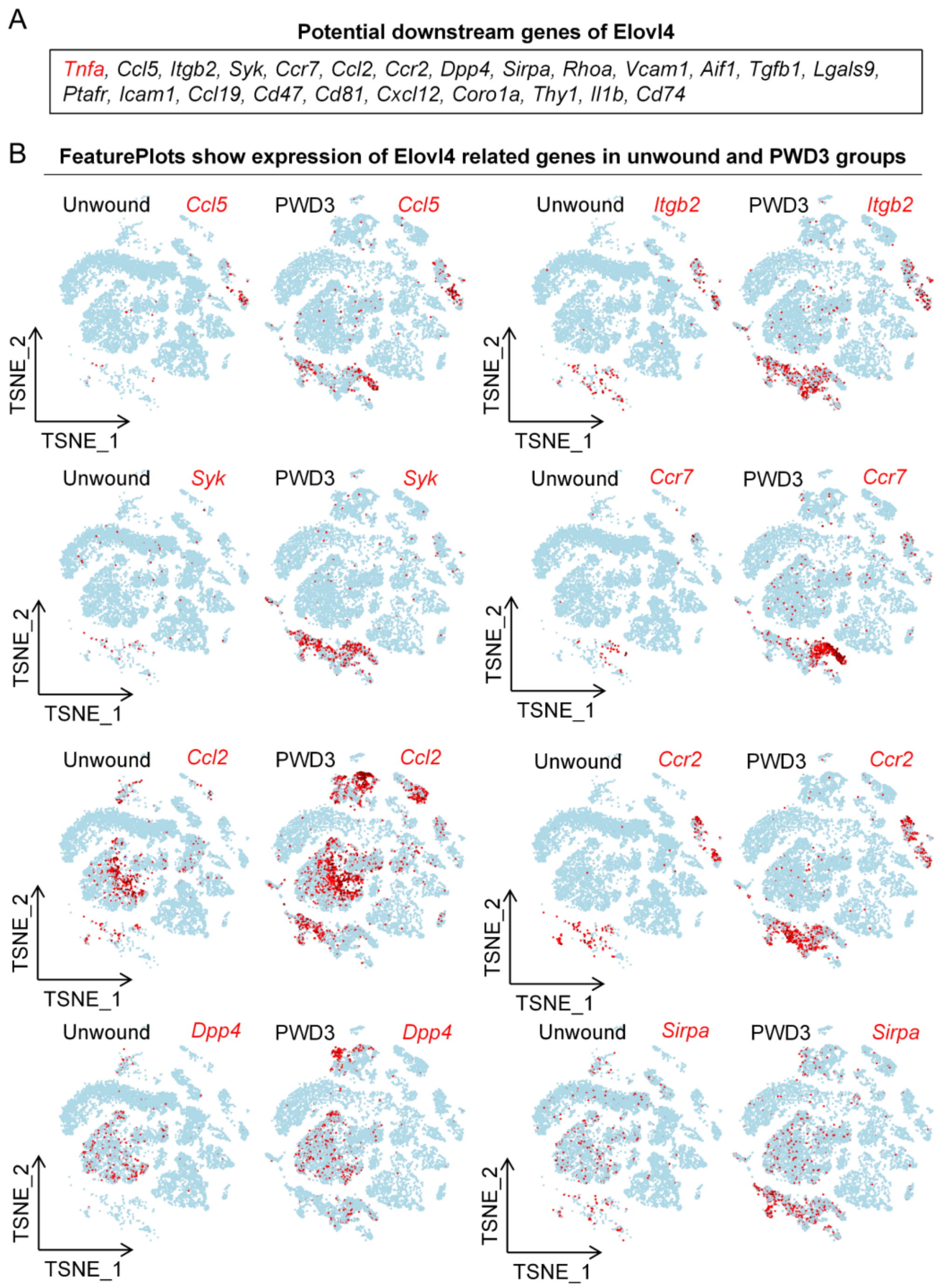

Figure S4. Downstream analysis of Elovl4.

A. A list of the top 5 ranked genes according to their P-values.

B. FeaturePlots show expression of Elovl4-related genes in unwound and PWD3 groups.

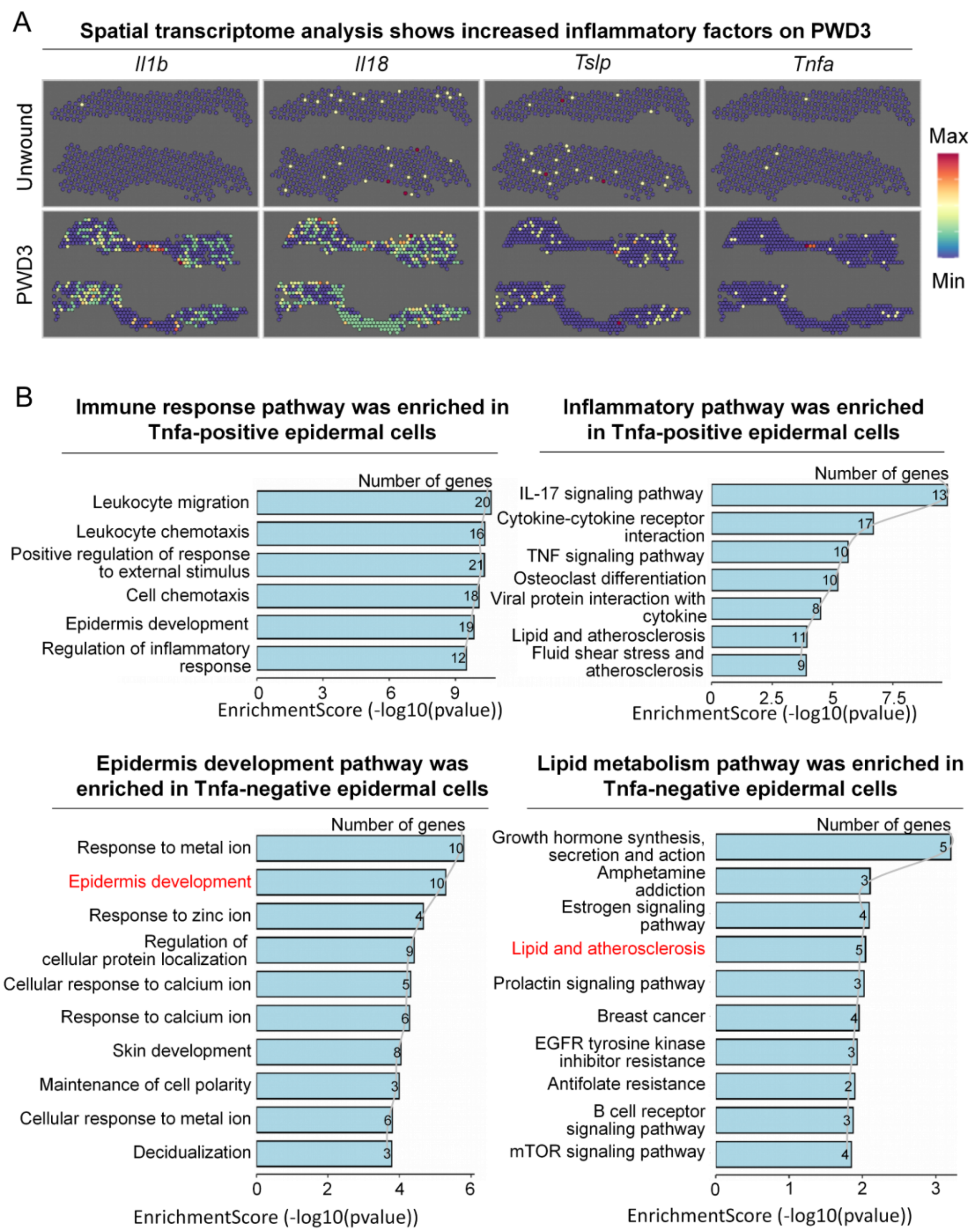

Figure S5. Identification of inflammatory factors.

A. ST-seq shows expression of inflammatory factors in unwound and PWD3 groups.

B. GO and KEGG analysis shows enriched immune response pathways in TNFα positive epidermal cells. Epidermis development and lipid metabolism are enriched in the TNFα-negative cells.

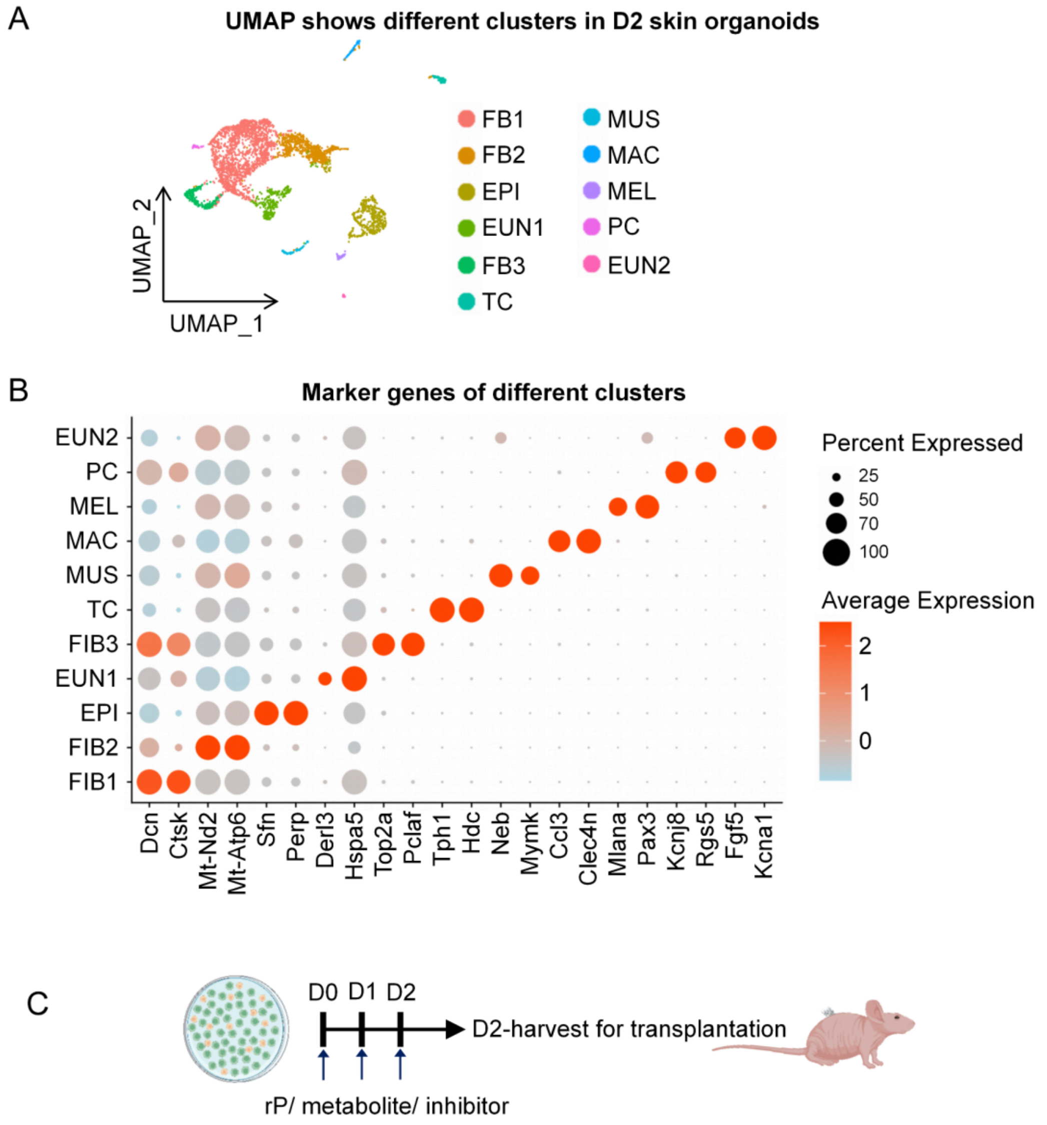

Figure S6. Single cell RNA-seq analysis of skin organoids.

A. UMAP shows different clusters in D2 skin organoids.

B. Dotplot shows marker genes of different clusters.

C. Schematic shows experimental design.

Figure S7. Bulk RNA-seq analysis of skin wound.

A

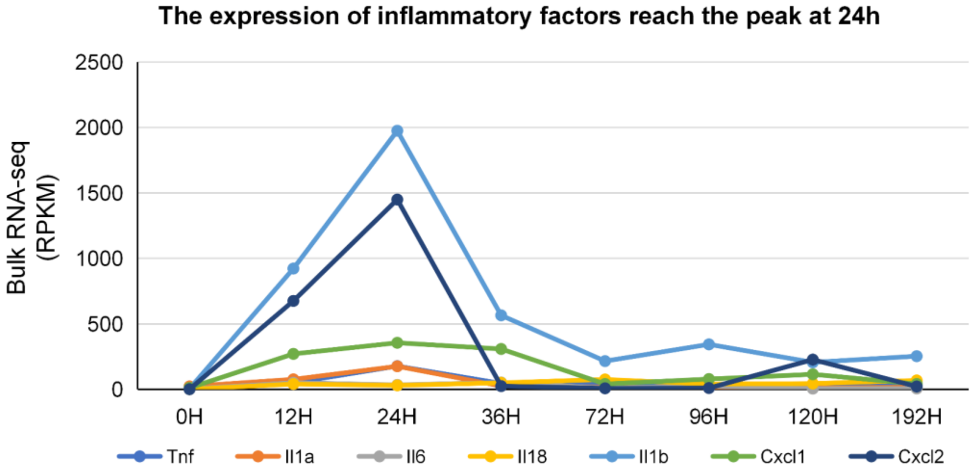

A. Line chart shows expressions of different inflammatory factors in PWD0-PWD8.
